# A Cretaceous origin for fire adaptations in the Cape flora

**DOI:** 10.1038/srep34880

**Published:** 2016-10-05

**Authors:** Tianhua He, Byron B. Lamont, John Manning

**Affiliations:** 1 Department of Environment and Agriculture, Curtin University, PO Box U1987, Perth, WA 6845, Australia; 2 Research Centre for Plant Growth and Development, School of Life Sciences, University of KwaZulu-Natal, Pietermaritzburg, Private Bag X01, Scottsville 3209, South Africa

## Abstract

Fire has had a profound effect on the evolution of worldwide biotas. The Cape Floristic Region is one of the world’s most species-rich regions, yet it is highly prone to recurrent fires and fire-adapted species contribute strongly to the overall flora. It is hypothesized that the current fire regimes in the Cape could be as old as 6–8 million years (My), while indirect evidence indicates that the onset of fire could have reached 18 million years ago (Ma). Here, we trace the origin of fire-dependent traits in two monocot families that are significant elements in the fire-prone Cape flora. Our analysis shows that fire-stimulated flowering originated in the Cape Haemodoraceae 81 Ma, while fire-stimulated germination arose in the African Restionaceae at least 70 Ma, implying that wildfires have been a significant force in the evolution of the Cape flora at least 60 My earlier than previous estimates. Our results provide strong evidence for the presence of fire adaptations in the Cape from the Cretaceous, leading to the extraordinary persistence of a fire-adapted flora in this biodiversity hotspot, and giving support to the hypothesis that Cretaceous fire was a global phenomenon that shaped the evolution of terrestrial floras.

Fire influences current global ecosystem patterns and processes^1^ and has had a major impact on plant evolution for at least 350 million years, a legacy that continues into many modern-day ecosystems^2^,^3^,^4^. The origin of fire-adapted lineages and the antiquity of current fire regimes are long-standing questions in evolutionary ecology. Fossil charcoal evidences are critical in identifying ancient fire activity but fossil data are rare or difficult to impossible to obtain in some regions. Fossil evidence may also be biased, as there are intrinsic taphonomic biases in the types of habitats most likely to preserve fossils. Fossils are preserved primarily in wetlands where fires seldom occur^5^. Thirdly, fossil evidence is necessary to validate the presence of ancient wildfire but not sufficient to indicate fire-driven evolution, as plants do not adapt to fire *per se* but rather to a particular fire regime.

Research to date shows that fire has driven the evolution of many plant traits in fire-prone environments, including whole-plant fire responses (resprouting after fire versus killed by fire but regenerating from stored seeds), serotiny (on-plant seed storage), and fire-stimulated seed germination and flowering^6^. As these traits are specific to fire-prone habitats, tracing the origin of such traits through studies on dated phylogenies can indicate when fire became sufficiently extensive and recurrent to select for fire-adapted traits. There is accumulating molecular evidence that fire-adapted traits go back many tens of millions of years^2^,^3^,^7^,^8^,^9^,^10^. Recently, some of these dates have been largely reconciled with fossil evidence. Using a dated phylogeny, Lamont and He^2^ traced the origin of fire-related seed storage in the ancient Gondwanan family Proteaceae to at least 76 Ma, and hypothesized that fire-prone communities were present in Australia by that time. New fossil evidence is consistent with these predictions showing that, for Proteaceae-dominated heathlands, charcoal was present in central Australia during the Late Cretaceous^11^. Further, Falcon-Lang *et al*.^12^ recently showed that the earliest fire-preserved *Pinus* fossil dated to 140−133 Ma while searching for evidence of fire in the early Cretaceous that might support the evolution of fire-related traits as postulated by He *et al*.^3^ to exist in *Pinus*. Thus, the approach of mapping ecological traits onto robust and dated phylogenies has become a credible tool for supplementing or even anticipating palaeontological information in understanding the development of fire-adapted biomes globally.

The Cape Floristic Region (hereafter The Cape) is one of the world’s most floristically diverse regions, with over 9 300 species of vascular plants in an area of just 90 760 km^2^, of which 70% are endemic^13^, yet the region is highly prone to recurrent fires and fire-exposed species contribute strongly to the overall flora^14^. Ecologists have long noted that the Cape ecosystems were not in equilibrium with the climate as fire prevented them reaching their ‘climax’ state, but it is only recently that fire has become accepted as shaping regional vegetation long before its use by humans^15^. The fossil record for South Africa is too poor to allow for confident reconstruction of past climates, vegetation or fire regimes^15^. Bytebier *et al*.^7^ used the evolutionary history of the orchid genus *Disa* to trace the origin of fire-stimulated flowering in some lineages and concluded that evolutionarily significant fire in the Cape may date back to *c.*18 Ma. This is considerably earlier than previous speculations of a late Miocene/Pliocene origin for fires in the Cape^6^ but much later than recent paleontological evidence for fire during the Lower Cretaceous in the eastern Cape^16^. The families Restionaceae, Proteaceae and Ericaceae were already present in the Arnot Pipe deposits at Banke, Namaqua, dated at 71−64 Ma^17^, and all are prominent and characteristic elements in the modern fire-prone Cape flora, pointing to a much earlier origin of fire-dependent communities in the Cape than currently accepted.

Here, we trace the origin of the key fire-dependent trait, fire-stimulated flowering (FSF), in the family Haemodoraceae. In association with resprouting, FSF takes advantage of the optimal resources and minimal competition for pollinators available immediately postfire^18^. The FSF response centers on the nature of cueing factors (e.g., ethylene), resource factors (e.g., extra nutrients) and contingency factors (e.g., season of burn)^18^. The evolutionary history of FSF has been explored recently in Orchidaceae, Proteaceae, Droseraceae and Loranthaceae and shown to date back to at least 50 Ma, indicating that the timing of flowering in many groups has had a long association with fire as an agent of natural selection^18^.

The Haemodoraceae is considered a Gondwanan family with its origin and one major vicariance event occurring before the separation of present Antarctica from South America−Africa^19^. Hopper *et al*.^19^ estimated the stem age of Haemodoraceae at 90 Ma, and 81 Ma for the divergence of the two subfamilies, Haemodoroideae and Conostylidoideae (Fig. 1). Based on the Hopper phylogeny, we assigned current distributions to all species in the phylogeny. Ancient range reconstruction indicated that the Haemodoraceae originated in southwestern Australia (SWA) 90 Ma (*P* = 0.69). The two subfamilies separated at 81 Ma (76–84, 95% highest posterior density, HPD), with the Conostylidoideae remaining in SWA (*P* = 1.00) and Haemodoroideae originating in South Africa (*P* = 0.78). *Haemodorum* migrated to eastern Australia 32 Ma, while the common ancestor of *Xiphidium* and *Schiekia* dispersed to South America at 30 Ma (Fig. 1). Ancestral state reconstruction suggests that the common ancestors of both Conostylidoideae and Haemodoroideae possessed FSF (*P* = 0.65 and 0.89 respectively). The stem of Haemodoraceae was reconstructed as FSF with *P* = 0.62 (Fig. 1). The appearance of FSF in Haemodoroideae 90 Ma suggests that a fire-prone environment existed in SWA at that time. This date is consistent with the 88 Ma previously reported from research on fire-proneness of Proteaceae in Australia^2^ and also reconciles with recent paleontological discoveries in Australia^11^. At this stage it is not clear how the ancestor of subfamily Haemodoroideae migrated to South Africa 81 Ma. The presence of FSF in the ancestor of Haemodoroideae at that time suggests that at least parts of the Cape must have already been fire-prone to allow this subfamily to establish there.

As fire-adaptations must be an evolutionary response at the community scale, we also traced the first appearance of another key fire-dependent trait, fire-stimulated germination (FSG) of seeds in the African Restionaceae. Fire-related heat shock, as well as chemicals in smoke and charred wood, provide cueing signals that trigger germination of seeds in fire-prone environments. FSG is adaptive as it enhances fitness by cueing germination to postfire conditions when light, water and nutrient resources are optimal^6^. Fire-stimulated seed germination has been reported for diverse Cape species, including members of the Restionaceae, Ericaceae and Proteaceae^20^. The nut-fruited Restionaceae species, for example *Cannomois virgata*, show marked improvement in germination on exposure to charate from fire^21^. Litsios *et al*.^22^ constructed a strongly supported phylogeny for Restionaceae, estimating the age of the root of the Restionaceae at 80 My and bifurcation of the Australian and Cape Restionaceae at 70 Ma (65–87, 95% HPD). For Cape Restionaceae, we collated research that tested seed germination as a response to fire cues (smoke, fire heat, charate). We then reconstructed the ancestral state for FSG of Cape Restionaceae. Both of the major lineages of the Cape Restionanceae were diagnostic for FSG (P = 0.89 and 0.67 respectively) (Fig. 2). The root of Cape Restionaceae was FSG with a posterior probability of 0.78, suggesting that the Cape has been fire-prone for at least 70 Ma.

Australian Restionaceae are deficient in studies on responses of seed germination to fire cues compared with the Cape, and it was not possible to reconstruct the ancestral state of the clade. Nevertheless, 13 species among ten genera spread throughout the clade (versus ~50 records for Cape Restionaceae) are reported to show FSG, suggesting an ancient origin of this trait in Australian Restionaceae as well. Coupled with morphological conservatism of the fruits and seeds in this family (mainly capsules with some nuts, like those in the Cape clade), and confirmed fire-proneness from at least 76 Ma in the region, it is reasonable to expect a matched physiology of seed germination for Australian Restionaceae as well. For example, *Baloskion tetraphyllum* has a capsule that responds to karrikin in smoke in a similar way to the effect of smoke on germination of Cape Restionaceae with capsules^23^. More research is needed on germination cues in Australian Restionaceae but the limited available evidence points to FSG at the root of that clade.

Our analysis suggests that wildfires have been sufficiently intense and reliable to initiate and maintain the evolution of fire-adapted plant traits from at least 81 Ma during the Cretaceous in the Cape of South Africa, as already demonstrated in Australia with a similar fire history. This is based on two monocot families with a much longer evolutionary history than the previous attempt with the orchid genus *Disa*^7^. These results reconcile with a recent report of burnt vegetation in the Eastern Cape in the early Cretaceous^18^. Elsewhere in the African continent, Afty *et al*.^24^ reported the presence of fire in Egypt 75 Ma. The Cretaceous is regarded as a ‘high-fire’ period in the Earth’s history^25^, with elevated atmospheric oxygen concentrations at this time^26^. However, Cretaceous fossil records are rare in the Southern Hemisphere^24^, leading some researchers to suggest that fire is a recent phenomenon in the Cape flora^5^,^7^,^15^. Our study helps redress the sparse studies on the history of fire in Africa relative to those for the northern hemisphere. Our results not only provide strong evidence for the support of fire adaptations in the Cape from the Cretaceous 81 Ma, leading to the extraordinary persistence of the Restionaceae and Haemodoraceae that are now significant elements in this biodiversity hotspot, but they also fill in a critical gap for support of the hypothesis that Cretaceous fire was a global phenomenon^25^.

A common drawback with using dated phylogenies to estimate the onset of fire-driven evolution is that it is dependent on the age of the study taxon. With an evolutionary history <20 My, *Disa* was not able to gauge the onset of fire older than 20 My. Using the family Proteaceae, with a 113-My evolutionary history, Lamont and He^2^ were able to estimate the origin of the fire-dependent proteoid lineage from rainforest ancestors as occurring some 25 My later. For Haemodoraceae, our analysis indicates that the Cape clade migrated there from SWA, implying that wildfires in the Cape must already have been sufficient to maintain their pre-existing, fire-dependent traits. Therefore, our method may still underestimate the age of fire in the Cape. Nevertheless, fire-dependent traits in two monocot families suggest significant fire there at least 81 Ma, only slightly younger than the estimate of 88 Ma from Proteaceae for Australia^2^ and 89 Ma for pines in the northern hemisphere^10^.

The Cape has a broadly Mediterranean-type climate. Along with the Mediterranean Basin, SWA, Central Chile, and California, these Mediterranean-type ecosystems constitute rare terrestrial biomes with extraordinary biodiversity. All five regions are variously prone to widespread crown fires^6^. A long-held view is that the typical Mediterranean vegetation developed since the Neogene, 25 Ma^27^, as former mesic, rainforest-clad environments became colder, drier and more fire-prone and favored the development of the classic, sclerophyllous shrub form^11^. Our analysis, together with other recent molecular phylogenetic studies^2^,^8^,^9^,^10^, indicate much older origins for some clades that are now prominent in fire-prone, Mediterranean-type habitats, suggesting that fire has been an important evolutionary force since the Mid- to Late Cretaceous, long before the Neogene. It is likely that the development of fire-prone systems was not controlled by global temperatures or climate seasonality, as currently, but rather by elevated atmospheric oxygen concentrations^26^. This would also explain the origin and continuing evolution of some fire-dependent traits in the Paleogene-Eocene (such as serotiny in *Banksia*^9^; resprouting in *Eucalyptus*^8^), as higher oxygen levels would allow much moister vegetation to burn^26^, and consequently serve to decouple the relationship between fire activity and fuel-moisture levels. Biomes in the Cape and Australia are much older than the advent of their Mediterranean-type climates suggest, sharing a much earlier fire-prone history that has shaped the evolution of their floras to explain their strongly-developed fire-dependence as observed today^28^,^29^.

## Methods

Dated phylogenies for the Haemodoraceae and Restionaceae were reconstructed from data used by Hopper *et al*.^19^ and Litsios *et al*.^22^. Both families were sampled extensively and the phylogenies were calibrated with commonly accepted fossils. For Haemodoraceae, we collated the current distribution and state of fire-stimulated germination (FSF) for each species from the literature^19^, inspection of web images (http://www.ispotnature.org/species-dictionaries/sanbi), expert advice, and our field observations. We first reconstructed the ancient distribution and determined the origin of the African clade. We further traced the origin of FSF in the South African clade of Haemodoraceae. We defined a (resprouting) species to have FSF if the flowering rate in the first one or two years was >twice as high as the subsequent years until the next fire. For Restionaceae, our analysis focused on the African clade. Litsios *et al*.^22^ showed that some genera are not monophyletic (e.g. *Restio* and *Cannomios*). We therefore accepted the phylogenetic position of the genera in the family where the majority of species clustered in the phylogenetic tree. We collated research that tested seed germination as a response to fire cues (smoke, fire-related heat or charate)^20^,^21^,^30^, and assigned the genus with the trait of fire-stimulated germination (FSG) if >80% of tested species showed a >25% and/or statistically significant improvement in seed germination when treated with fire cues; otherwise the genus was assigned as “unknown”. FSG data for 50 species spread throughout the African clade were available.

### Ancestral geographic distribution reconstruction in Haemodoraceae

Four regions were recognized in this analysis: Cape, South Africa; SWA; Eastern Australia; and South America, based on Hopper *et al*.^19^. A Markov chain Monte Carlo (MCMC) binary method was used to construct the ancestral geographic distribution of Haemodoraceae, implemented with RASP^31^. We used the Haemodoraceae maximum credible tree and regional occurrence as input, and set the state frequencies to be fixed (Jukes-Cantor Model) and the among-site rate variation as Gamma (+G). We set 1,000,000 MCMC generations with the first 10% as burn-in.

### Ancestral state reconstruction

Based on the well-calibrated and supported maximum credible phylogenetic trees of Haemodoraceae and Restionaceae and trait matrices for FSF and FSG, we used MultiState in BayesTraits^32^ to determine the most likely ancestral traits at each node for FSF in Haemodoraceae, and FSG in African Restionaceae. We employed a Bayesian framework using MCMC sampling to search for optimal parameter estimates. The MCMC parameter searches consisted of 1,000,000 iterations with 20,000 as burn-in. We used maximum likelihood parameter estimates as starting values in the MCMC analyses.

## Additional Information

**How to cite this article**: He, T. *et al*. A Cretaceous origin for fire adaptations in the Cape flora. *Sci. Rep.* **6**, 34880; doi: 10.1038/srep34880 (2016).

## Figures and Tables

**Figure 1:**
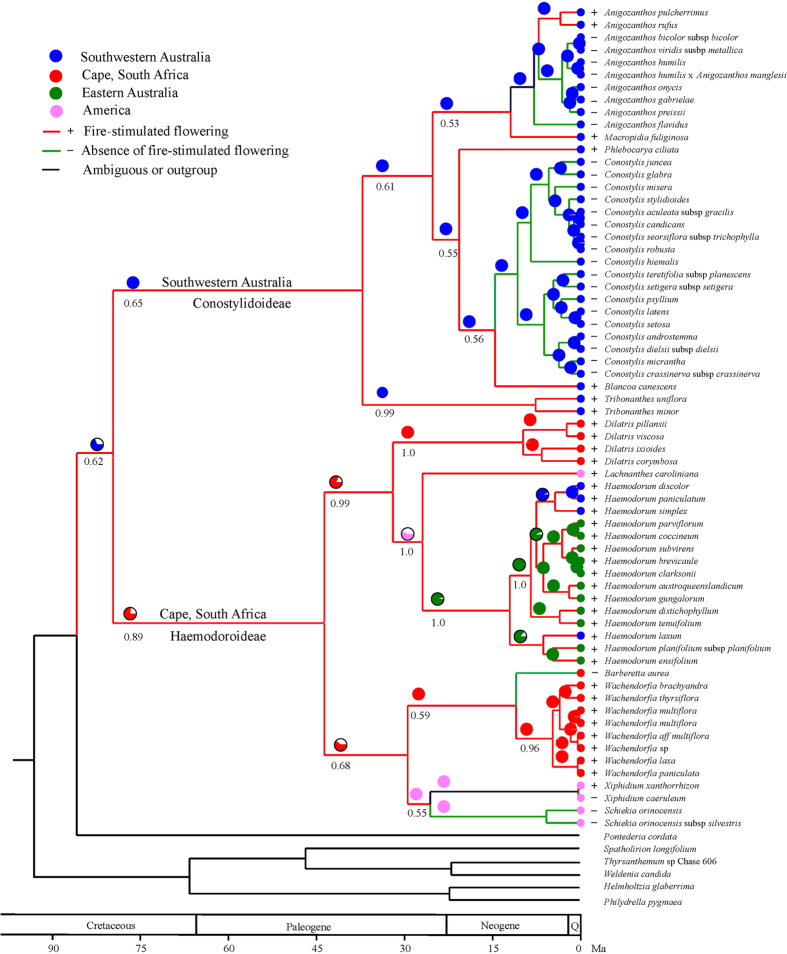
Reconstruction of ancient geographic range and ancestral state of fire-stimulated flowering in Haemodoraceae. Numbers below branches indicate the posterior probability of the node possessing fire-stimulated flowering.

**Figure 2:**
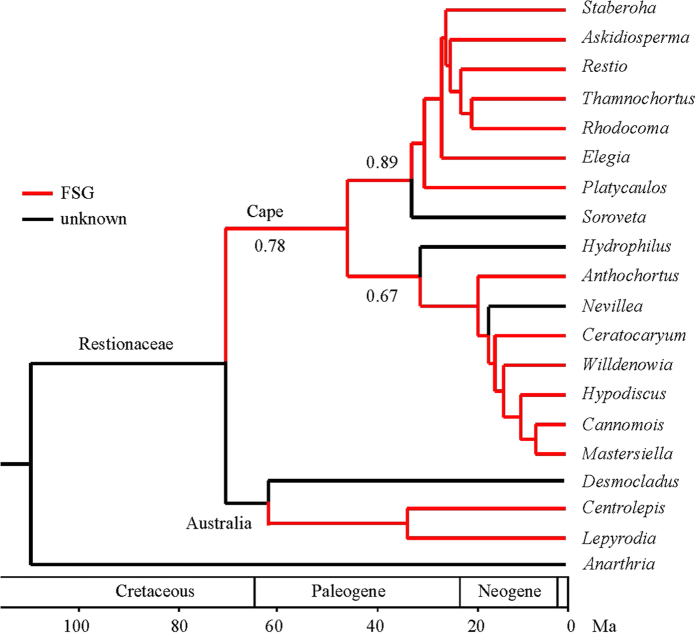
Reconstruction of ancestral state of fire-stimulated germination of seeds in the Cape Restionaceae. Numbers below branches indicate the posterior probability of the node possessing fire-stimulated germination of seeds.
